# Fluctuations in Intracellular CheY-P Concentration Coordinate Reversals of Flagellar Motors in *E. coli*

**DOI:** 10.3390/biom10111544

**Published:** 2020-11-12

**Authors:** Yong-Suk Che, Takashi Sagawa, Yuichi Inoue, Hiroto Takahashi, Tatsuki Hamamoto, Akihiko Ishijima, Hajime Fukuoka

**Affiliations:** 1 Graduate School of Frontier Biosciences, Osaka University, 1-3 Yamadaoka, Suita, Osaka 565-0871, Japan; cheyong@fbs.osaka-u.ac.jp (Y.-S.C.); tatsuki.hamamoto@oist.jp (T.H.); ishijima@fbs.osaka-u.ac.jp (A.I.); 2 Institute of Multidisciplinary Research for Advanced Materials, Tohoku University, Aoba-ku, Sendai 980-8577, Japan; t.sagawa@sigma-koki.com (T.S.); y.inoue@sigma-koki.com (Y.I.); hiroto.takahashi.b7@tohoku.ac.jp (H.T.)

**Keywords:** chemotaxis, signal transduction, diffusion, response regulator, CheY, flagellar motor, *E. coli*

## Abstract

Signal transduction utilizing membrane-spanning receptors and cytoplasmic regulator proteins is a fundamental process for all living organisms, but quantitative studies of the behavior of signaling proteins, such as their diffusion within a cell, are limited. In this study, we show that fluctuations in the concentration of the signaling molecule, phosphorylated CheY, constitute the basis of chemotaxis signaling. To analyze the propagation of the CheY-P signal quantitatively, we measured the coordination of directional switching between flagellar motors on the same cell. We analyzed the time lags of the switching of two motors in both CCW-to-CW and CW-to-CCW switching (∆τ_CCW-CW_ and ∆τ_CW-CCW_). In wild-type cells, both time lags increased as a function of the relative distance of two motors from the polar receptor array. The apparent diffusion coefficient estimated for ∆τ values was ~9 µm^2^/s. The distance-dependency of ∆τ_CW-CCW_ disappeared upon loss of polar localization of the CheY-P phosphatase, CheZ. The distance-dependency of the response time for an instantaneously applied serine attractant signal also disappeared with the loss of polar localization of CheZ. These results were modeled by calculating the diffusion of CheY and CheY-P in cells in which phosphorylation and dephosphorylation occur in different subcellular regions. We conclude that diffusion of signaling molecules and their production and destruction through spontaneous activity of the receptor array generates fluctuations in CheY-P concentration over timescales of several hundred milliseconds. Signal fluctuation coordinates rotation among flagella and regulates steady-state run-and-tumble swimming of cells to facilitate efficient responses to environmental chemical signals.

## 1. Introduction

Diffusion of signaling proteins within a cell and their interaction with the molecules responsible for the input and output of the signal play an essential role in all biological signal transduction systems [1,2,3]. In both prokaryotes and eukaryotes, cells use transmembrane receptors to sense environmental stimuli and to generate intracellular signals in the form of messenger molecules. These intracellular messenger molecules diffuse through the cytoplasm to their targets, where they regulate cellular functions such as gene expression and locomotion. In an extracellular example, neurotransmitters released from the presynaptic membrane diffuse across the synaptic cleft to receptors embedded in the postsynaptic membrane to initiate action potentials. Therefore, diffusion of signaling molecules plays an important role in both intracellular and extracellular sensory transduction. However, quantitative studies of behavior in signaling systems including diffusion of signaling molecules are limited because the input-output relationship in signaling pathways is difficult to measure directly with high temporal and spatial resolution. Precise knowledge of the in vivo kinetics and localization of the reactions that generate and degrade signals, and of the diffusion parameters for signaling proteins, are essential to characterize sensory input-output pathways fully.

Chemotaxis enables *Escherichia coli* cells swimming in a liquid environment to track and navigate chemical gradients with high precision [4,5]. *E. coli* uses chemoreceptor proteins, located primarily at the cell pole(s), to detect specific chemicals and to monitor pH and temperature. The receptors employ a His-Asp two-component phosphorelay to transmit sensory messages to the flagellar motors [4]. *E. coli* cells swim by rotating their left-handed helical flagellar filaments: counter-clockwise (CCW) rotation produces forward swimming; clockwise (CW) rotation triggers random turning movements, called tumbles. The chemoreceptors assemble in large signaling arrays connected by two cytoplasmic proteins: CheA, a histidine autokinase, and CheW, a scaffolding protein that couples CheA activity to receptor control. Receptor arrays modulate the autophosphorylation activity of CheA to control the flux of phosphoryl groups from CheA to the response regulator CheY. Phospho-CheY (CheY-P) is the intracellular messenger that binds to a flagellar motor to induce CW rotation [6,7,8,9,10]. CheY-P molecules appear to reach the flagellar motors through intracellular diffusion [1]. CheZ, a dedicated CheY-P phosphatase, degrades the CW signal, but it is located mainly at the polar receptor arrays through interaction with a variant form of CheA [11,12]. Thus, CheY-P is both generated and degraded at receptor arrays, but CheY-P molecules that escape from CheZ in the array encounter additional, although less concentrated, CheZ molecules as they diffuse through the cytoplasm.

Previously, we demonstrated that under steady-state conditions with no external stimuli, two flagellar motors on the same *E. coli* cell coordinately switch their rotational direction [13]. The switching time lag (∆τ_correlation_) of two coordinated motors depended on their relative distance from the receptor array at the cell pole. In addition, we showed that binding and dissociation of CheY-P molecules at the motors triggered CW and CCW rotation episodes, respectively [6]. To explain these results, we proposed that increases and decreases in CheY-P concentration directly and coordinately regulate the rotational direction of motors under steady-state conditions. The ∆τ_correlation_ represents the delay in the propagation of CheY-P concentration changes to motors that are positioned at different distances from the receptor array.

In the present study, to clarify the mechanism of the intracellular signaling of *E. coli* under steady-state, we asked how motor coordination changes when CheZ is uniformly distributed throughout the cell rather than localized at the polar receptor array. Our working hypothesis predicts that the propagation of increases and decreases in the CheY-P concentration depends on the distance from the polar receptor array, owing to the diffusion of CheY and CheY-P in the cytoplasm, and the rates of phosphorylation and dephosphorylation of CheY by polarly localized CheA and CheZ. To test this model, we first demonstrated that, in a wild-type cell, the ∆τ for coordinated switching between two motors (either CCW-to-CW or CW-to-CCW) depends on the relative distance between the two motors and their distance from a polar receptor array. In contrast, there was no distance-dependency for ∆τ of CW-to-CCW switching when CheZ was distributed uniformly throughout the cytoplasm. Moreover, using photorelease of serine from a caged compound, we found that, when CheZ is uniformly distributed, there was no distance-dependency in the response to an instantaneously applied attractant signal. These results are consistent with the predictions of a simulation based on the localization of the CheZ phosphatase and the diffusion of CheY and CheY-P molecules in the cytoplasm. We conclude that the increases and decreases in CheY-P concentration are generated by spontaneous bursts of receptor array activity and that the rotational direction of multiple flagellar motors is coordinated under steady-state conditions. This signal fluctuation depends on the diffusion coefficient of the signaling molecule and on the signal-generating and signal-destroying reactions in the polar receptor array. We propose that the steady-state fluctuations in CheY-P concentration thus serve to regulate run-and-tumble swimming of the cell through coordinated switching of the flagellar motors. Thus, an *E. coli* cell would maintain the chemotaxis system on high alert to respond efficiently to environmental chemical signals rather than conserve energy by keeping the system in a resting mode.

## 2. Materials and Methods

### 2.1. E. coli Strains, Plasmids, and Cell Growth

The strains and plasmids are listed in Table S1. All strains were derived from the K12 strain RP437, which is wild-type for chemotaxis [14]. The replacement of the wild-type cheA gene with cheA(M98L) to make CheA_S_^−^ cell [15], the replacement of the wild-type fliC gene with the fliC-sticky gene [16], and the replacement of the wild-type cheZ gene with cheZ(F98S) [11] were carried out using the λ red recombinase and tetracycline sensitivity selection method [17,18]. LB broth (1% bactotryptone (BD, Sparks, MD, USA), 0.5% yeast extract (BD, Sparks, MD, USA), 0.5% NaCl (Wako, Osaka, Japan)) was used for culture growth, transformations, and plasmid isolation. Tryptone broth (TB) (1% bactotryptone, 0.5% NaCl) was used to grow cells for measurements of motor rotation. Growth conditions were described in Supplemental Methods. For all measurement, the cells were suspended in 10NaMB (10 mM potassium phosphate buffer (Wako, Osaka, Japan), pH 7.0; 0.1 mM EDTA-2K (Wako, Osaka, Japan), pH 7.0; 10 mM NaCl, 75 mM KCl (Wako, Osaka, Japan)).

### 2.2. Measurement of Rotation of Multiple Flagellar Motors

Cells were prepared and measured by a method similar to that described in our previous report [13] (see also Supplementary Methods). For measurement of rotation of the motor, a polystyrene bead, diameter (φ) 0.5 µm (Polysciences; Warrington, PA, USA), was attached to the sticky flagellar filaments [16]. The sticky filaments made by this mutant FliC readily adsorbed polystyrene beads without modification of beads. The phase-contrast images of beads through the objective lens (UPlanFl 40× NA 0.75 Ph2; Olympus, Tokyo, Japan) were recorded with a high-speed charge-coupled device (CCD) camera (IPX-VGA210LMCN; Imperx, Boca Raton, FL, USA) at 1250 or 1255 frames/s. This high-speed CCD camera was controlled by the measurement software Real Time Video Nanometry (RTVN), which we developed using LabVIEW 2009 (National Instruments, Austin, TX, USA) [19]. Images of bead were fitted by a two-dimensional Gaussian function for every sampling frame, and the position of a bead was expressed as the peak position of X and Y coordinates of a fitted Gaussian curve. The bead position was approximated by an ellipse function every 500 frames, and the bead position was corrected to approximate a perfect circle centered on the origin. The rotation angle was calculated for every two sampling frames, and a time trace of rotational velocity and rotational direction were estimated by repeating this process every video frame.

To observe the location of GFP-CheW, a blue laser beam (wavelength, 488 nm) (Sapphire 488-20-SV; Coherent, Hercules, CA, USA) was focused on the back focal plane of the objective lens. After recording the bead rotation with the high-speed CCD camera, the fluorescence image of GFP-CheW and the phase-contrast images of the bead and cell were recorded at 120 frames/s with a second CCD camera (DMK23G618; The Imaging Source, Bremen, Germany). The distance between the fluorescent focus at the cell pole derived from the GFP-CheW fluorescence and the rotational center of the rotating bead was measured and defined as the distance from the receptor array to motor.

### 2.3. Measurement of the Response Time to the Instantaneously Applied Photoreleased Serine

Cell preparation, the microscopic system, and the conditions for photoreleasing serine from caged serine were the same as in our previous study [19] (Figure S3; see also Supplementary Methods). Caged serine surrounding an *E. coli* cell in a microscopic field was photolyzed by irradiation with a violet laser beam (wavelength, 405 nm) (KBL-90C-A; Kimmon Koha, Tokyo, Japan, or OBIS 405-50 LX; Coherent, Hercules, CA) for 80 ms. The violet laser beam was uniformly applied to the irradiated area (diameter, 32 μm), and the energy density of the laser beam in the irradiated area was 370 mW·mm^−2^. The rate constant for the photolysis of caged serine in the irradiated area was 0.16 s^−1^. When RTVN detected the switching of the flagellar motor from the CCW to CW, it opens a mechanical shutter (UHS1 ZM 2; Uniblitz, San Diego, CA, USA) positioned in front of the laser beam for 80 ms. The distance between the polar receptor array and the flagellar motor was quantified by the polar localization of GFP-CheW and the rotational center of the rotating bead as described above.

### 2.4. Correlation Analysis

To analyze the correlation in the switching between flagellar motors, the rotational velocity was classified into three states by the following procedure. The time-trace of the rotational velocity was filtered by the Chug-Kennedy filtering algorithm (C-K filter) [20], using an analytical window of 100 data-points and a weight of 10. Rotational velocities of more than +20 Hz, between +20 Hz and −20 Hz, and less than −20 Hz were assigned as CCW rotation (+1), pause (0), and CW rotation (−1), respectively. The correlation analysis was performed by applying Equation (1) to the time traces of the rotational directions between two flagellar motors:

(1) $$Z\left(\tau \right)=\frac{\frac{1}{N}\sum_{t=1}^{N} [x\left(t\right)\cdot y\left(t+\tau \right)-\bar{x\left(t\right)}\cdot \bar{y\left(t\right)}]}{\sqrt{\frac{1}{N}\sum_{t=1}^{N}{ [x\left(t\right)-\bar{x\left(t\right)}]}^{2}}\cdot \sqrt{\frac{1}{N}\sum_{t=1}^{N}{ [y\left(t\right)-\bar{y\left(t\right)}]}^{2}}}$$

where *Z* is the function used for the correlation analysis, *t* is time, τ is the time difference, *N* is the total number of sampling points, and *x*(*t*) and *y*(*t*) are the time traces of the rotational directions of two motors, respectively. In cells producing GFP-CheW, this function was applied to the motor closer to the fluorescent focus at one of the cell poles. We analyzed motors on the cells less than 3 µm long. All correlations *Z* (τ) were calculated (−1 ≤ *Z* ≤ 1) by Equation (1) using the traces for 1 or 2 min.

### 2.5. Estimation of Δτ_correlation_, ∆τ_CCW-CW_, and ∆τ_CW-CCW_

To analyze the time difference of switching between two motors from correlation analysis, the near 0-sec peak determined by correlation analysis was fitted by a Gaussian function, and the peak time of the fitted curve was defined as Δτ_correlation_. ∆τ_CCW-CW_ and ∆τ_CW-CCW_ were analyzed as follows. In cells for which coordinated switching between two motors was detected, all the coordinated switching events were extracted from a trace, and the time difference of switching (∆τ) between 2 different motors was calculated for every extracted switching event. The time difference was calculated for both CCW-to-CW switching (∆τ_CCW-CW_) and CW-to-CCW switching (∆τ_CW-CCW_), respectively. Their average values were plotted against [M2^2^-M1^2^], where M1 and M2 are the distances from the polar chemoreceptor array to the proximal and distal motors, respectively. The apparent diffusion coefficients for CCW-to-CW and CW-to-CCW switching signals were estimated from the slope of the approximation line to the plots for ∆τ_CCW-CW_ and ∆τ_CW-CCW_, respectively. To estimate the diffusion coefficient for CW-to-CCW switching, only plots having a plus value were used.

### 2.6. Simulation of the Change in the Intracellular Concentration of CheY-P and the Estimation of Response Time for Photoreleased Serine

To investigate the effect of polar localization of CheZ on changes in the CheY-P concentration, we performed a particle-based simulation. In the simulation, a 2 µm-long and 0.8 µm-wide rectangle was assumed, and 9000 CheY particles were initially placed randomly within this rectangle. These particles could diffuse freely in two dimensions independently of each other. The particles were reflected by each wall. The step size of a particle every time-interval was calculated from the following equations:

(2) $$dx=\sqrt{2\times D\times \Delta t}$$

(3) $$dy=\sqrt{2\times D\times \Delta t}$$

where *dx* and *dy* are the step sizes of the particle in each time increment, *D* is the diffusion coefficient, and ∆*t* is the time-interval of the calculation. In the calculation, 0.002 ms was used as ∆*t*, and 11.7 µm^2^/s [13] was used as the diffusion coefficient of the CheY and CheY-P molecules.

When the receptor array is in an inactive state, CheA is not phosphorylated. On the other hand, when the receptor array is in an active state, CheA is auto-phosphorylated at the constant rate k_1_ in the reaction scheme shown as follows:

(4) $$A\overset{k_{1}}{\to }AP$$

where *A* is non-phosphorylated CheA and AP is auto-phosphorylated CheA (CheA-P). We used 30 s^−1^ (k_1_) as a value close to the past report [2]. The number of CheA-P was counted every ∆*t*. Phosphorylation of CheY and dephosphorylation of CheY-P occur in the reaction scheme as follows:

(5) $$Y+AP\overset{\;k_{2}\;}{\to }YP+A$$

(6) $$YP+Z\overset{\;k_{3}\;}{\to }Y+Z$$

where *YP* is CheY-P and *Z* is CheZ. *k*_2_ and *k*_3_ are the rate constants for CheY phosphorylation and CheY-P dephosphorylation, respectively. In all simulations, *k*_2_ was 1.0 × 10^8^ M^−1^ s^−1^ as previously reported [2]. The value of *k*_3_ was changed according to the calculation (see each figure and figure legend). In all simulations, 4500 CheA molecules and 3200 CheZ molecules were used to approximate physiological conditions [21]. In the simulation for the cell in the presence of CheZ localization (the CheA_S_^+^ cell), all 4500 CheA molecules were placed at the left edge of the rectangle (within 20 nm from the edge). Probability of phosphor-transfer to each CheY particle (P_YP_) in this area was calculated as follows:

(7) $$P_{YP}=k_{2}\cdot [AP]\cdot \Delta t$$

where [*AP*] is the concentration of CheA-P estimated from the number of CheA-P by assuming a 0.02 µm-long, 0.8-μm wide, and 0.8 µm high volume located at the left edge of rectangle. In total, 2600 CheZ molecules were placed at the left edge of the rectangle, corresponding to their binding to 2600 CheA_S_ molecules at cell pole (21), while 600 CheZ molecules were placed at the bulk-cytoplasmic area of the rectangle. The probability of dephosphorylation of each CheY-P particle at the left edge (P_dephos-Y@pole_) and cytoplasm (P_dephos-Y@cyto_) were calculated as follows:

(8) $$P_{dephos-Y@pole}=k_{3}\cdot { [Z]}_{pole}\cdot \Delta t$$

(9) $$P_{dephos-Y@cyto}=k_{3}\cdot { [Z]}_{cyto}\cdot \Delta t$$

where [*Z*]_pole_ is the concentration of CheZ at the cell pole estimated from the number of CheZ by assuming a 0.02 µm-long, 0.8-µm wide, and 0.8 µm high volume is located at the left edge of the rectangle. [*Z*]_cyto_ is the concentration of CheZ at the cytoplasm estimated from the number of CheZ by assuming a 1.98 µm-long, 0.8-µm wide, and 0.8 µm high volume is located at the cytoplasmic area of the rectangle. Thus, CheY was phosphorylated at the left edge of the rectangle, and CheY-P was rapidly dephosphorylated at the left edge of the rectangle and dephosphorylated more slowly in the remainder of the rectangle.

In the simulation for the cell in the absence of CheZ localization (the CheA_S_^−^ cell), all CheA molecules were again located at the left edge of this rectangle, but all 3200 CheZ molecules were placed at the cytoplasmic area of the rectangle. Thus, all CheY molecules were again phosphorylated within 20 nm from the left edge of the rectangle, but CheY-P molecules were dephosphorylated throughout the whole rectangle. The P_YP_ and the P_dephos-Y@cyto_ was estimated for each CheY or CheY-P particle and for every Δt by the same method as described above. In both simulations, assuming CheA_S_^+^ and CheA_S_^−^ cells, CheY molecules were phosphorylated from 0 to 1 s, and CheY-P was dephosphorylated constantly from 0 to 2 s. The motors were positioned at 0.05, 0.2, 0.4, 0.6, 0.8, 1.0, 1.2, 1.4, 1.6, and 1.8 µm from the left edge of the rectangle. A 0.2 µm-long and 0.8 µm-wide rectangle spanning the larger rectangle was centered on each motor, and the number of CheY-P molecules within this area was counted. The number of molecules was converted to the concentration by assuming a 0.2 µm-long, 0.8-µm wide, and 0.8 µm high volume. The simulations were performed at least 10 times for each value of k_3_ with both the CheA_S_^+^ cell and the CheA_S_^−^ cell. The response time for CW to CCW rotation to instantaneously applied attractant stimulus was estimated from the average of the traces at each value of k_3_ as the time required for the CheY-P concentration to fall below the threshold concentration of 3.2 µM for CW-to-CCW switching after the CheA activity was inhibited [22].

## 3. Results

### 3.1. Coordination of Flagellar Motors under Steady-State Conditions

**Figure 1:**
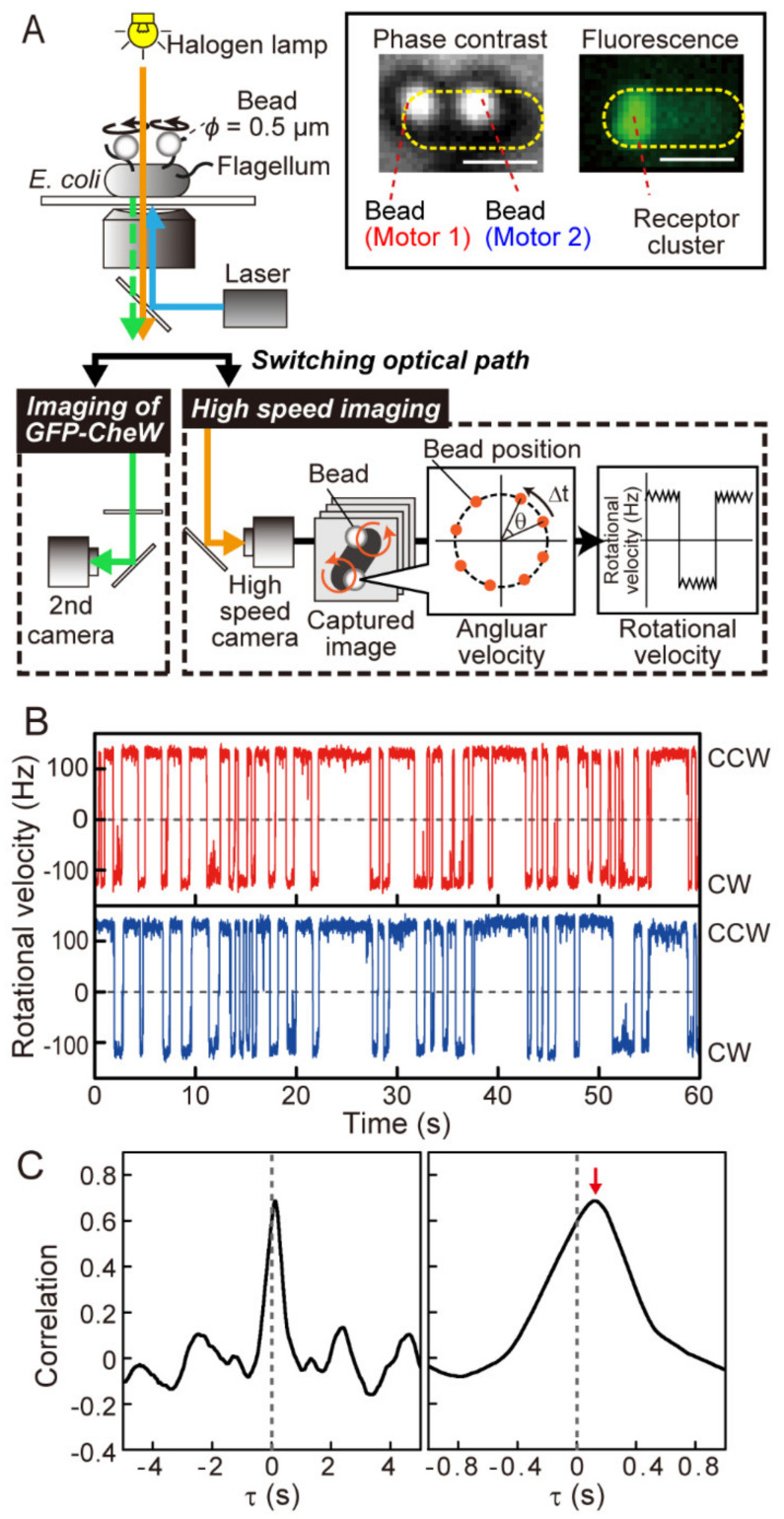
Coordination of steady-state switching of flagellar motors on a wild-type (CheA_S_^+^) cell. (**A**) Schematic diagram of the measurement system. The cell was stuck to a coverslip, and polystyrene beads (φ = 0.5 µm) were attached to the sticky flagellar stubs to calculate the angular velocity of the motor from the position of each bead. The phase-contrast image of each bead was recorded with a high-speed charge-coupled device (CCD) camera (1250 or 1255 frames/s). A time trace of rotational velocity and rotational direction of the motor was calculated from the bead position in each sampling frame. The fluorescence image was recorded with a second CCD camera by switching optical path. Phase-contrast images of the cells and beads (left inset) and fluorescence imaging of the cells (right inset) are also shown. Bar, 1 µm. The yellow-dotted ellipses indicate the cell bodies, and the positions of motors 1 and 2 are approximated from the position of the beads to which they are attached. The position of the polar receptor array was determined by the localization of GFP-fusion of CheW (GFP-CheW). (**B**) The time traces of the rotational velocities of motors 1 (red: proximal to the receptor array) and 2 (blue; distal from receptor array) on a wild-type (CheA_S_^+^) cell. The plus and minus values represent counterclockwise (CCW) and clockwise (CW) rotations, respectively. (**C**) Cross-correlation profile between the time traces of motors 1 and 2, which are depicted in (**B**) (left), with a magnified version of it (right). The analysis was based on proximal motor 1. Correlations were calculated using Equation (1), as shown in the Materials and Methods. The red arrow indicates the time of peak correlation (∆τ_correlation_ = +0.121 s).

We monitored the rotation of two motors on the same cell with small beads attached to their flagellar filaments, as illustrated in Figure 1A (see also Materials and Methods). The cells expressed GFP-CheW to determine the position of their chemoreceptor array relative to the two motors. The rotational motion of each bead attached to a flagellar stub was followed with a high-speed CCD camera to obtain a time-trace of the rotational velocity and directional switching of the two motors [13]. Two motors on a single wild-type cell coordinately switched their rotational directions, both from CCW to CW and from CW to CCW (Figure 1B). A correlation analysis for the time traces of motor 1 and motor 2 (see Materials and Methods) showed a major peak near 0 s, indicating a sub-second time delay between switching of the two flagellar motors (Figure 1C). These results are consistent with our previous report [13].

### 3.2. Coordination between Flagellar Motors in the Absence of Polar Localization of CheZ

**Figure 2:**
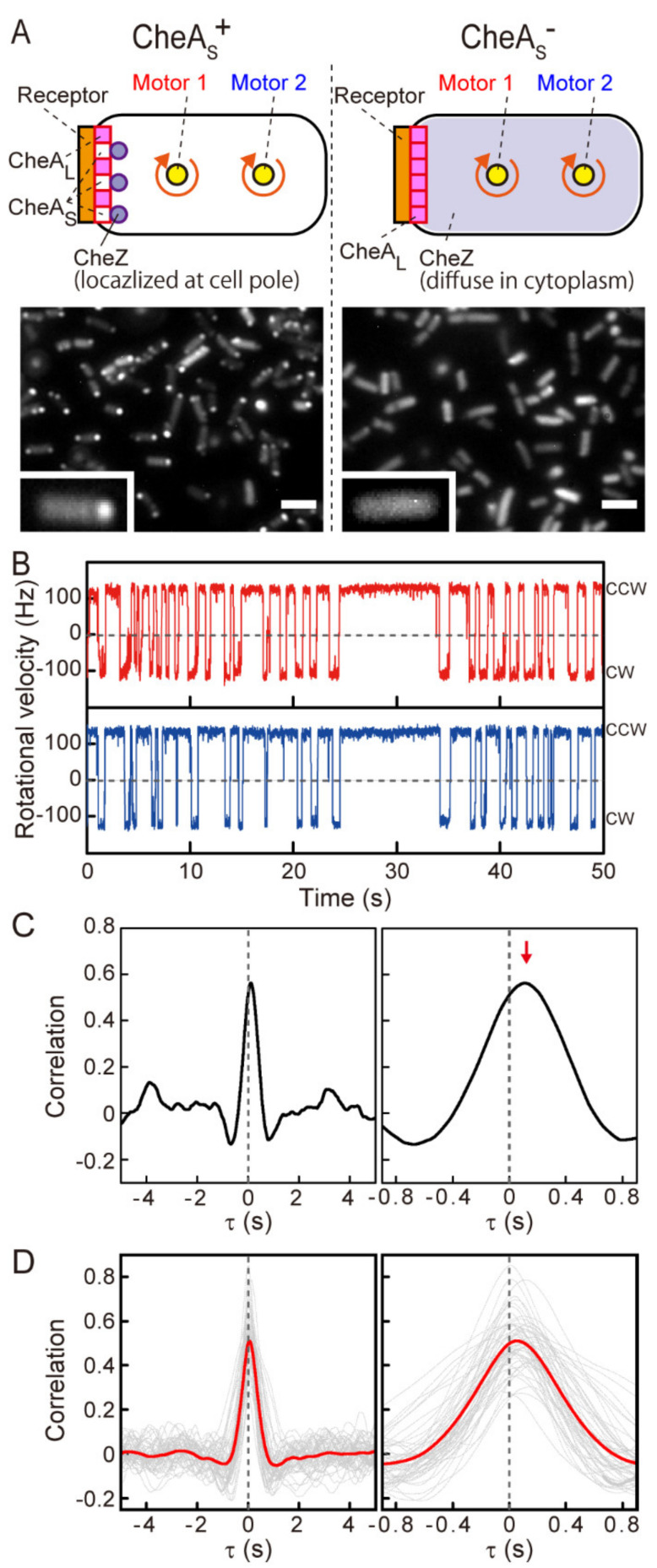
Coordination of steady-state switching of flagellar motors on a CheA_S_^−^ cell. (**A**) Localization of CheZ-EGFP in a CheA_S_^+^ cell (left) and a CheA_S_^−^ cell (right). A magnified image of each strain is shown at the bottom. Bar = 3 µm. (**B**) The time traces of the rotational directions of the proximal motor 1 (red) and the distal motor 2 (blue) on a CheA_S_^−^ cell. (**C**) A cross-correlation profile of the time traces of motors 1 and 2, which are depicted in (**B**) (left) and magnified (right). The analysis was based on proximal motor 1. The red arrow indicates the time of peak correlation (∆τ_correlation_ = +0.110 s). (**D**) Gray lines indicate a correlation analysis for CheA_S_^−^ cells measured at 1250 fps. The traces of 45 cells that showed coordination of switching are shown. The analysis was performed on the cells with monopolar or bipolar localization of GFP-CheW. The average of the Δτ_correlation_ was 68 ± 73 ms (mean ± SD). In a monopolar cell, the correlation was calculated based on the motor closer to receptor array. In a bipolar cell, the correlation was calculated based on the motor closer to more brighter receptor array. The red line shows the average traces of the correlation analyses from 45 cells.

To investigate whether localization of CheZ phosphatase activity affects the signaling process, we compared the coordination of motor switching in cells that produce both full-length CheA (CheA_L_) and the N-terminally truncated form of CheA known as CheA short (CheA_S_) with cells that produce CheA_L_ only (Figure 2A). In wild-type cells, a large fraction of CheZ localizes at the chemoreceptor array through a reversible interaction with CheA_S_ [11,12], an alternate translation product of the *cheA* gene initiated at codon ATG-98 [23,24]. The CheA_S_ protein is not essential for overall chemotaxis [25], and its expression can be eliminated by mutations that alter *cheA* codon 98. In this work, we used mutant strains encoding CheA-M98L to eliminate CheA_S_ synthesis [15] (Table S1). In these strains, CheZ should be uniformly distributed in the cytoplasm, with none being concentrated at the receptor array [11]. Accordingly, CheY-P dephosphorylation should occur throughout the cytoplasm instead of predominantly at the cell poles [12]. We confirmed the expected localization patterns of CheZ by imaging CheZ-GFP in CheA_S_^+^ and CheA_S_^−^ cells (Figure 2A). Approximately 85% of the CheA_S_^+^ cells exhibited polar localization of CheZ-GFP. In contrast, almost all of the CheA_S_^−^ cells exhibited a uniform cytoplasmic distribution of CheZ-GFP.

Like the motors on CheA_S_^+^ cells (Figure 1B), motors on CheA_S_^−^ cells coordinately switched their rotational directions, both from CCW to CW and from CW to CCW (Figure 2B). A correlation analysis of the rotational directions of the proximal motor 1 and the distal motor 2 showed a major peak near 0 s, indicating a sub-second time delay between switching of the two flagellar motors (Figure 2C). This sub-second coordination prevailed in 84/86 cells (98%) that we observed (Figure 2D, 45/45 cells measured at 1250 fps and Figure S1, 39/41 cells measured at 1255 fps) and was still apparent in the averaged correlation profile (Figure 2D and Figure S1, red lines). These results indicate that multiple flagellar motors coordinately switch their rotational direction in both CheA_S_^+^ and CheA_S_^−^ cells under steady-state conditions in the absence of chemoeffector stimuli. Therefore, polar localization of CheZ is not essential for steady-state coordination of motor switching.

### 3.3. Distance-Dependent Time Lags in Steady-State Motor Switching

**Figure 3:**
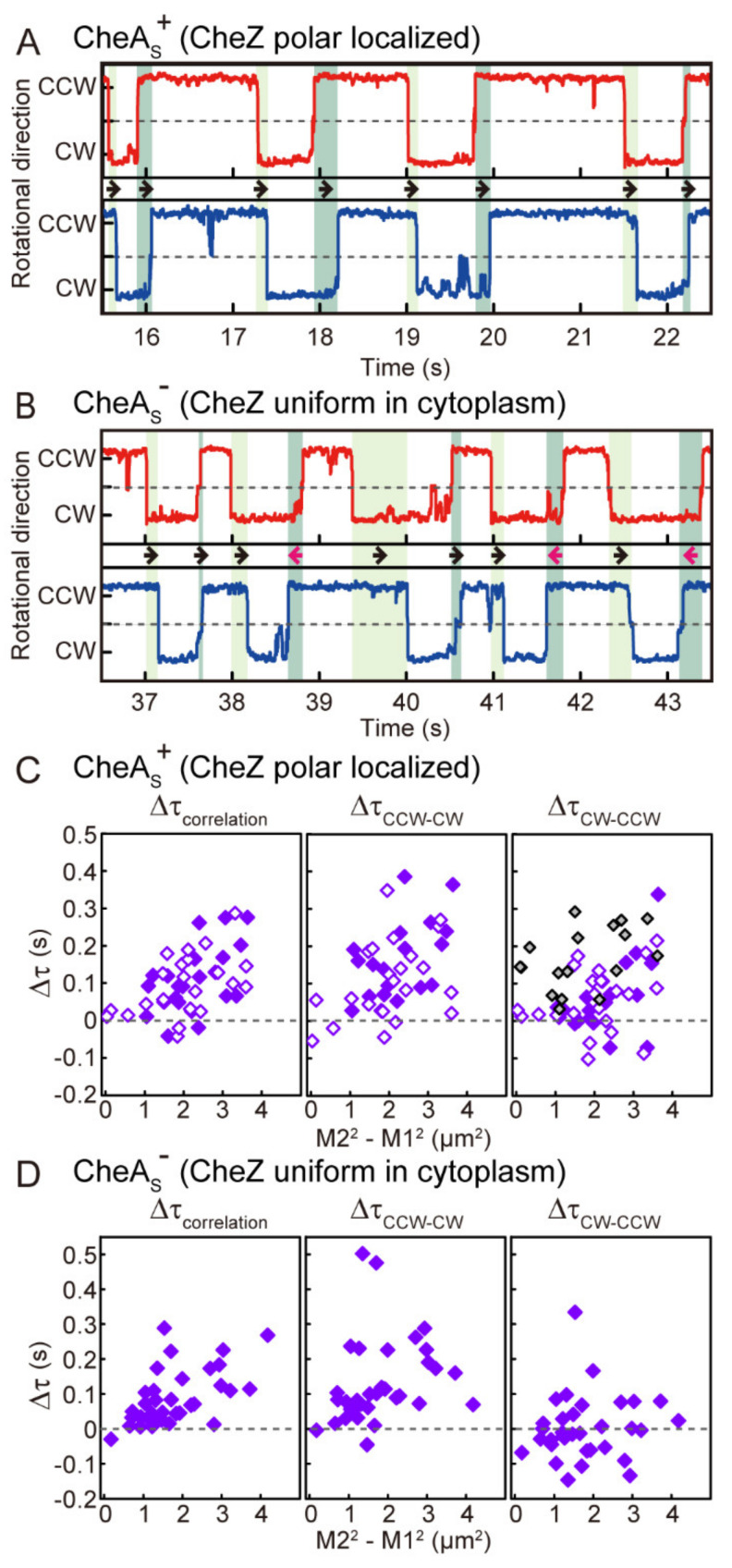
Time lags between switching of the two motors as a function of the distance from the polar chemoreceptor array. (**A**) The time traces of the rotational directions of the proximal motor 1 (red) and the distal motor 2 (blue) of a CheA_S_^+^ cell, shown in Figure 1B, over a short time period. Light-green and dark-green hatched areas indicate the time lags of switching between the two motors from CCW-to-CW and CW-to-CCW, respectively. Forward arrows indicate that switching of motor 1 preceded that of motor 2. ∆τ_CCW-CW_, ∆τ_CW-CCW_, and M2^2^-M1^2^ values of this cell are 0.16 s, 0.03 s, and 1.21 µm^2^, respectively. (**B**) The time traces of the rotational directions of the proximal motor 1 (red) and the distal motor 2 (blue) of a CheA_S_^−^ cell, shown in Figure 2B, over a short time period. Colored hatches are as in (**A**). Forward arrows indicate that switching of motor 1 preceded that of motor 2, and reverse arrows indicate that the switching of motor 1 was delayed relative to switching of motor 2. ∆τ_CCW-CW_, ∆τ_CW-CCW_, and M2^2^-M1^2^ values of this cell are 0.231 s, −0.026 s, and 1.26 µm^2^, respectively. (**C**) The relationship between the ∆τ value and [M2^2^-M1^2^] for CheA_S_^+^ cells. Closed purple squares show the plots measured in this study (n = 22 cells). Open purple squares show the plots estimated in our previous study [13] (n = 22 cells in ∆τ_correlation_, and n = 19 cells in ∆τ_CCW-CW_ and ∆τ_CW-CCW_). The data were reused from reference [13] with permission. Gray squares show the relationship between the ∆τ value and [M2^2^-M1^2^] in CheA_S_^+^ cells in which CheA_S_ was overproduced (n = 17 cells). Spearman’s rank correlation coefficient for ∆τ_correlation_, ∆τ_CCW-CW_ and ∆τ_CW-CCW_ were 0.49, 0.53, and 0.36, respectively. For the estimation of the coefficient for Δτ_CW-CCW_, the data of the cell that CheAs is overexpressed was excluded. (**D**) The relationship between the ∆τ value and [M2^2^-M1^2^] for CheA_S_^−^ cells (n = 33 cells). Spearman’s rank correlation coefficient for ∆τ_correlation_, ∆τ_CCW-CW_, and ∆τ_CW-CCW_ were 0.61, 0.42, and -0.05, respectively. (**A**–**D**) To evaluate the propagation of CheY-P precisely, cells with a monopolar localization of GFP-CheW were chosen for analysis. The correlation analyses were made based on the motor closer to the polar chemoreceptor array.

Although motor switching events were nearly synchronous, close inspection of the rotation traces in a CheA_S_^+^ cell revealed consistent time lags between switching of the two motors (Figure 3A; corresponding to the longer time traces in Figure 1B). The motor closer to the polar chemoreceptor array (proximal motor) always switched before the motor farther from the array (distal motor). This held true for both CCW-to-CW and CW-to-CCW switching (Figure 3A, light- and dark-green hatches, black arrows). Magnified rotational time traces for a CheA_S_^−^ cell also revealed time lags between motor switching events. For CCW-to-CW switching, the motor closer to the chemoreceptor array (Figure 3B, red trace) preceded the motor that was farther from the array (Figure 3B, blue trace), as in CheA_S_^+^ cells. However, in contrast to the CheA_S_^+^ case, CW-to-CCW switching of the motor closer to the receptor array did not always precede the switching of the more-distant motor in CheA_S_^−^ cells. For example, the proximal motor (motor 1) preceded the distal motor (motor 2) in one switching event (Figure 3B, dark-green hatches and black arrows), but motor 2 preceded motor 1 in another switching event (Figure 3B, dark-green hatches and magenta arrows). In 55% of CW-to-CCW switching, motor 1 delayed to motor 2 in this cell.

To quantify these switching behaviors, we analyzed the relationship between the distance of each motor from the polar receptor array and the time difference in the onset of switching, which was estimated from the peak time of the correlation profile (∆τ_correlation_). The ∆τ_correlation_ values were plotted against [M2^2^-M1^2^], where M1 and M2 are the distances from the polar chemoreceptor array to the proximal and distal motors, respectively. In both CheA_S_^+^ and CheA_S_^−^ cells, the aggregate ∆τ values scaled with [M2^2^-M1^2^] (Figure 3C,D, left).

Next, we analyzed the relation between ∆τ and [M2^2^-M1^2^] for CCW-to-CW switching and for CW-to-CCW switching (Figure 3A,B, light- and dark-green hatched area; see Materials and Methods). For CCW-to-CW switching, the ∆τ_CCW-CW_ values for both CheA_S_^+^ and CheA_S_^−^ cells scaled with [M2^2^-M1^2^] (Figure 3C,D, middle). For CW-to-CCW switching in CheA_S_^+^ cells, the ∆τ_CW-CCW_ values also scaled with [M2^2^-M1^2^] (Figure 3C, right, closed, and opened purple). However, CheA_S_^−^ cells exhibited both positive and negative ∆τ_CW-CCW_ values evenly distributed around 0 s; there was no correlation with the corresponding [M2^2^-M1^2^] values (Figure 3D, right).

To confirm that the different CW-to-CCW switching behavior of the CheA_S_^−^ cells arose from a uniform cytoplasmic distribution of CheZ, we repeated the switching experiments with CheA_S_^+^ cells that had a mutant form of CheZ. The CheZ-F98S protein has CheY-P phosphatase activity, but cannot bind to CheA_S_ [11]. The behavior of these cells was comparable to that of CheA_S_^−^ cells (Figure S2).

These results indicate that, in CheA_S_^+^ cells, the intracellular signals that induce both CCW-to-CW and CW-to-CCW switching events emanate at the polar receptor arrays and propagate to the flagellar motors by diffusion, accounting for the distance-dependent time delay in motor switching responses. We estimated the apparent diffusion coefficient for CCW-to-CW signals and for CW-to-CCW signals in CheA_S_^+^ cells from the slopes of linear fits to the ∆τ versus distance data (Figure 3C, middle and right). The values (9.3 ± 2.7 and 9.6 ± 0.6 µm^2^/s (mean ± SD), respectively) were in agreement with estimates obtained in previous studies [1,13,19].

We note that not all CheA_S_^+^ cells exhibited distance-dependency of ∆τ values in CW-to-CCW switching (Figure 3C, right). Because loss of polar localization of CheZ in CheA_S_^−^ cells abolishes the distance-dependency of the switching times, we thought that the anomalous CheA_S_^+^ cells might have a more uniform cytoplasmic distribution of CheZ. Indeed, ~15% of CheA_S_^+^ cells did not show polar localization of CheZ (Figure 2A). Moreover, plasmid-encoded expression of CheA_S_ at elevated levels in CheA_S_^+^ cells restored the distance-dependency of ∆τ values for CW-to-CCW switching in all cells that were examined (Figure 3C, right, gray).

### 3.4. The Effect of Polar Localization of CheZ on Distance-Dependent Motor Response Times to an Instantaneously Applied Chemoattractant

To determine whether the coordinated CW-to-CCW switching in steady-state is induced by a decrease in CheY-P concentration, we measured the response of CheA_S_^−^ cells to an instantaneously applied serine signal, which was photoreleased from caged serine (Figure S3). The area surrounding the targeted cell (φ = 32 µm) was irradiated with a violet laser (φ = 405 nm, 370 mW mm^−2^) to release free serine. In this experiment, the cellular response was measured in the presence of 1 mM caged serine. The concentration of photoreleased serine reached 10.6 µM during 80 ms of laser irradiation, and the serine concentration decreased at sub-seconds, because of diffusion, after laser irradiation was terminated [19].

**Figure 4:**
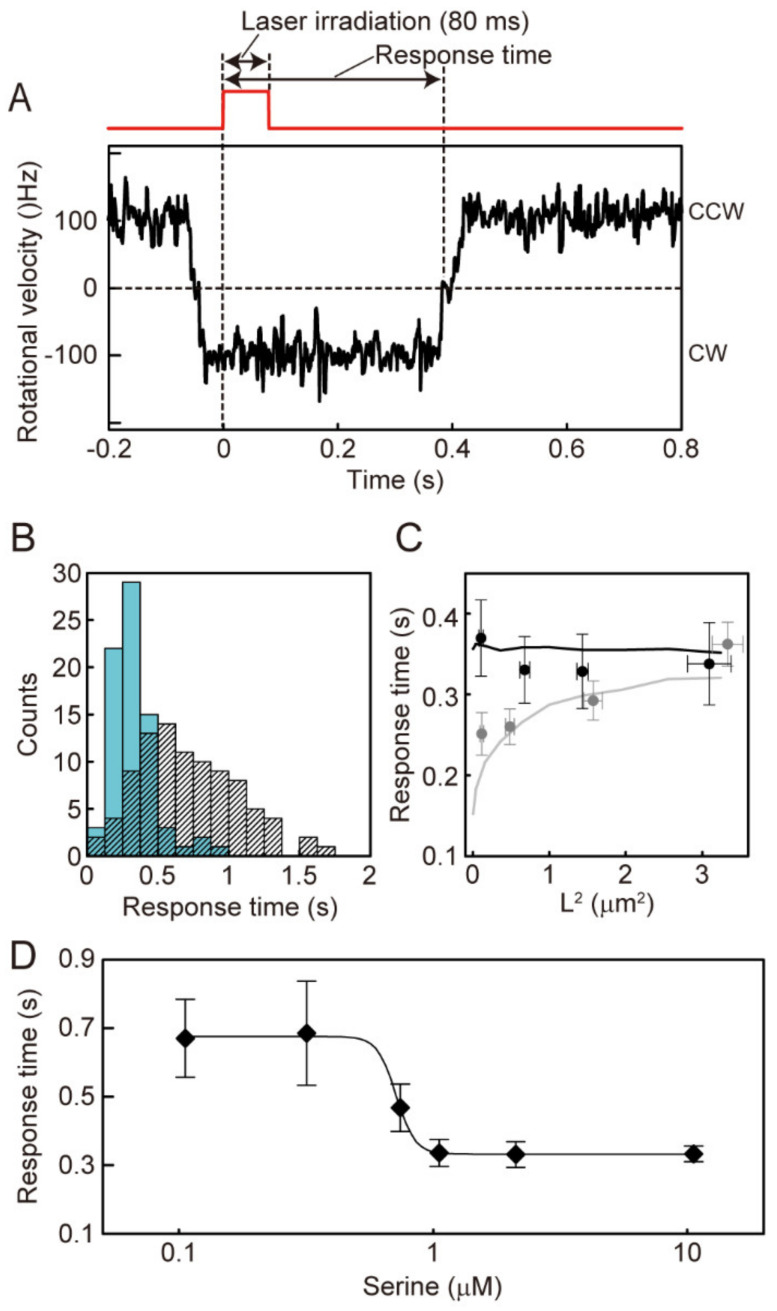
Measurement of the response to an instantaneously applied attractant stimulus of photoreleased serine. (**A**) Typical response of a CheA_S_^−^ cell after laser irradiation in the presence of 10.6 µM released serine (1000 µM of caged serine was contained in motility buffer). The laser shutter was open at 0 s for 80 ms (red line) immediately following a CCW-to-CW switching event. The interval between the initiation of laser irradiation and the first CW-to-CCW switch was defined as the response time. (**B**) Histogram of the response time of CheA_S_^−^ cells that exhibit monopolar and bipolar localization of GFP-CheW. Blue bars show the histogram of the response times obtained from 76 cells exposed to 10.6 µM released-serine. Gray hatched bars delineate the histogram of the CW duration obtained from 96 trials for 76 cells in the presence of 1000 µM caged serine without laser irradiation. (**C**) The relationship between the response time and the square of the distance from the polar receptor array to motor (L^2^). Black plots show the relationship for CheA_S_^−^ cells that show monopolar localization of GFP-CheW. Error bars show the standard error of the mean. The response times in the presence of 10.6 µM released-serine are shown (n = 45 cells). The black line is the estimation of the response time from the simulation shown in Figure 5C when the rate constant for dephosphorylation of CheY-P by CheZ (k_3_) is 1.6 × 10^6^ M^−1^ s^−1^. The experimentally measured response time of CheA_S_^−^ cells was well fitted by this value of k_3_. Gray plots show the relationship for CheA_S_^+^ cells that show monopolar localization of GFP-CheW (the data were reused from reference [19] with permission). The gray line is the estimation of the response time from the simulation shown in Figure 5B when k_3_ is 4.0 × 10^6^ M^−1^ s^−1^. The experimentally measured response time of CheA_S_^+^ cells was well fitted by this value of k_3_. (**D**) Relationship between the response time of CheA_S_^−^ cells and the concentration of released-serine. The response times for 0.11 (n = 9 cells), 0.32 (n = 9 cells), 0.74 (n = 9 cells), 1.1 (n = 11 cells), 2.1 (n = 12 cells), and 10.6 (n = 44 cells) µM released serine are shown. The black line shows the fitted curve using the Hill equation. Error bars show the standard error of the mean.

When the Tsr chemoreceptor binds serine, CheY-P production is inhibited by the suppression of CheA autophosphorylation, and the CheY-P concentration is decreased by CheZ. As CheY-P concentration decreases, the fraction of CheY-P molecules bound to FliM decrease, and the rotational direction of the motor reverses from CW to CCW. To investigate whether the CW-to-CCW switching is induced by a decrease in CheY-P concentration, the shutter shielding the laser beam from the cells was opened in response to CCW-to-CW switching of a targeted motor (serine was photoreleased following CCW-to-CW switching) (Figure 4A, red line). We then measured the response time, which was defined as the duration of CW flagellar rotation immediately after laser irradiation (Figure 4A). The response time includes five successive events: (1) the time required for serine molecules to diffuse to and become bound by Tsr; (2) the time required for inactivation of CheA activity after serine binds to Tsr; (3) the time required for the CheY-P concentration to be decreased by the phosphatase activity of both array-localized CheZ (mostly), bulk-cytoplasmic CheZ (rarely), and diffusion of CheY-P in CheA_S_^+^ cells, or the time required for CheY-P concentration to be decreased by bulk-cytoplasmic CheZ and diffusion of CheY-P in CheA_S_^−^ cells; (4) the time required for CheY-P to dissociate from the motor; (5) the time required for switching from CW-to-CCW rotation within the CheY-P-depleted motor.

A typical result is shown in Figure 4A. The shutter was opened at 0 s for 80 ms, and the direction of flagellar rotation switched to CCW 396 ms later (Figure 4A). In the presence of photoreleased serine, the average response time of CheA_S_^−^ cell was 329 ± 162 ms (Figure 4B, blue). In the presence of caged serine without laser irradiation, the average CW duration immediately following TTL (Transistor-transistor-logic) from the A/D (Analog/Digital) converter was 793 ± 514 ms (Figure 4B, black; see Materials and Methods). These results were consistent with the response of CheA_S_^+^ cells reported previously [19,26]. Thus, the duration of CW rotation immediately following laser irradiation was also significantly shortened by photoreleased serine in CheA_S_^−^ cells. Therefore, the response time is a reliable parameter to assess the response time of CheA_S_^−^ cells to serine.

Next, we compared the relation between the square of the distance from the polar receptor array to each motor (L^2^) and the response times of CheA_S_^+^ and CheA_S_^−^ cells. In our previous work, we showed that the response time of CheA_S_^+^ cells depended on L^2^ (Figure 4C, gray plots) [19]. On the other hand, the response time of CheA_S_^−^ cells was independent of L^2^ (Figure 4C, black plots). These results indicate that, in CheA_S_^+^ cells, the polar localization of CheZ delays the decrease in CheY-P concentration depending on the distance from the receptor array, whereas in CheA_S_^−^ cells, the CheY-P concentration decreases at the same rate throughout the cell because CheZ is distributed uniformly throughout the cytoplasm of the cell.

To investigate the effect of polar localization of CheZ on the sensitivity of the cellular response to serine, the relationship between the response time and the concentration of photoreleased serine was compared in the presence and absence of polar localization of CheZ. In CheA_S_^−^ cells, the average CW duration after photorelease of serine was ~670 ms when the released-serine concentration was <0.32 µM, whereas a response time of ~330 ms was observed when the released-serine concentration was >1.0 µM (Figure 4D). The K_1/2_ estimated from the Hill equation was 0.72 ± 0.01 µM (mean ± SE), which is 4.3-fold higher than that of CheA_S_^+^ cells (0.17 ± 0.11 µM) [19]. This result is consistent with a previous report of the higher phosphatase activity of CheZ in the presence of CheA_S_ in vitro [27]. Therefore, polar localization of CheZ enhances the sensitivity for serine. A similar tendency was reported for cells expressing CheZ-F98S, which does not localize to the cell pole, by detecting ensemble FRET from the cell population [12]. This higher sensitivity to serine would be due to the higher phosphatase activity of CheZ assembled into receptor array in CheA_S_^+^ cells, as described in the next section. A higher sensitivity for serine would be useful for detecting and migrating within shallow gradients at low serine concentrations.

### 3.5. Simulation of the Change in CheY-P Concentration and Response Time in the Presence and Absence of Polar Localization of CheZ

**Figure 5:**
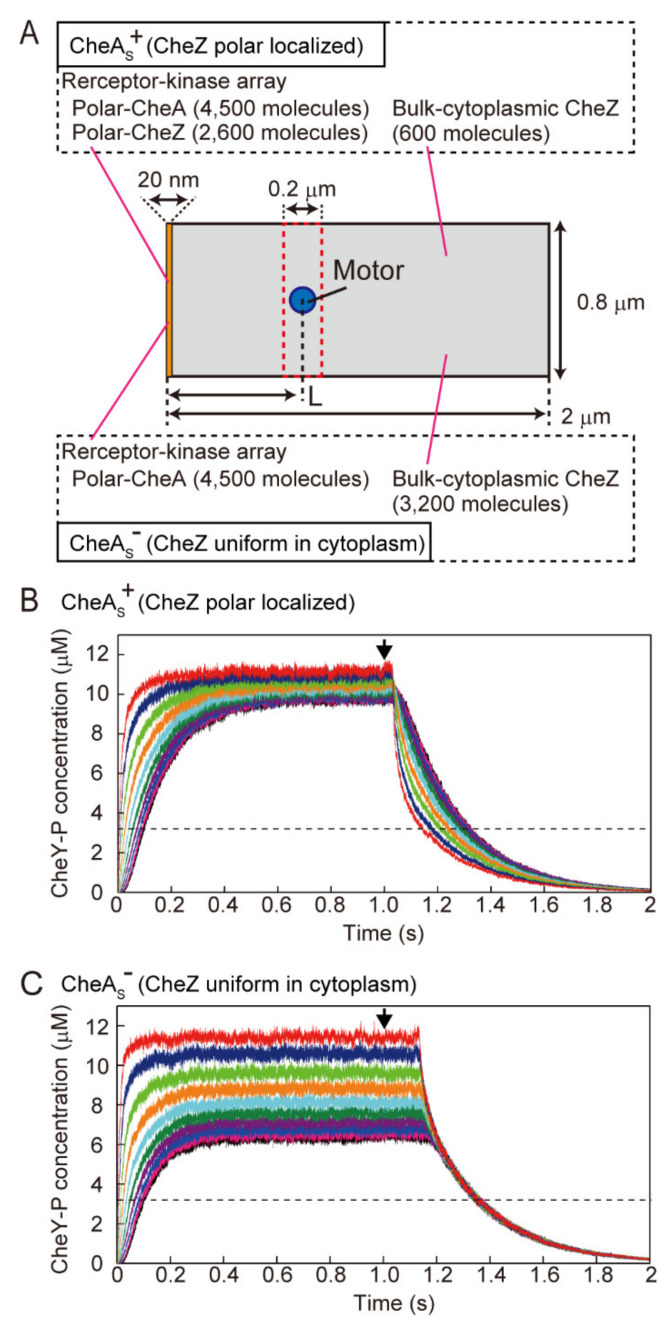
Simulation for the change in CheY-P concentration in the presence and absence of polar localization of CheZ. (**A**) A scheme depicting the method used to estimate the CheY-P concentration around a flagellar motor. Distance between the polar receptor-kinase array and motor shown as L. The number of CheY-P molecules within a 0.2-µm-long and 0.8-µm-wide area surrounding a motor (red dotted area) was counted and was converted to the concentration (see Materials and Methods). (**B**) Simulation for a CheA_S_^+^ cell when the rate constant for dephosphorylation of CheY-P (k_3_) of polar localized CheZ was 4.0 × 10^6^ M^−1^ s^−1^. Distances between the cell pole and the motor are, in µm, 0.05 (red), 0.2 (dark blue), 0.4 (green), 0.6 (orange), 0.8 (cyan), 1.0 (green), 1.2 (violet), 1.4 (blue), 1.6 (magenta), and 1.8 (black), respectively. At each position, the average trace from 13 simulations is shown. (**C**) Simulation for a CheA_S_^−^ cell when the k_3_ value for cytoplasmic CheZ is 1.6 × 10^6^ M^−1^ s^−1^. Distances from the cell pole and colors are the same as for the CheA_S_^+^ cell. At each position, the average trace from 10 simulations is shown. The results of the simulation using other k_3_ values are shown in Figure S4. In both simulations, the activity of CheA was turned on at 0 s, and a plateau level of CheY-P was reached within 1 s. CheA activity was turned off at 1 s (downward arrow).

To discuss the distance-dependency of time lags of switching between two motors (∆τ_CCW-CW_ and ∆τ_CW-CCW_) in CheA_S_^+^ cells and the lack of distance-dependency of ∆τ_CW-CCW_ in CheA_S_^−^ cells, we modeled the change in CheY-P concentration by simulating the diffusion of CheY and CheY-P molecules and the area of phosphorylation of CheY and of dephosphorylation of CheY-P, respectively. In a simulated CheA_S_^+^ cell, CheY and CheY-P molecules diffuse two-dimensionally in a rectangle (Figure 5A, top). Each CheY molecule is phosphorylated within a narrow area at the left side of the rectangle (the cell pole), and each CheY-P molecule is mostly dephosphorylated within the same area (see Materials and Methods). In this simulation, both the increase and decrease in CheY-P concentration showed delays dependent on distance from the cell pole, where the phosphorylation of CheY and the dephosphorylation of CheY-P occur (Figure 5B and Figure S4C). In the simulated CheA_S_^−^ cell, CheY is phosphorylated at the cell pole, whereas CheY-P is dephosphorylated in the bulk-cytoplasmic area at the same rate regardless of distance from the polar receptor array (Figure 5A, bottom; see Materials and Methods). In this situation, the increase in CheY-P concentration showed a delay dependent on the distance from the cell pole, similar to the CheA_S_^+^ case. However, CheY-P concentration decreased at the same rate independent of distance from the cell pole (Figure 5C and Figure S4D). Therefore, the distance-dependency of ∆τ_CCW-CW_ and ∆τ_CW-CCW_ in coordinated switching in wild-type cells can be explained by fluctuations in CheY-P concentration, taking into consideration the area of phosphorylation of CheY and dephosphorylation of CheY-P and the diffusion of CheY and CheY-P molecules.

To address the distance-dependency of serine response times in CheA_S_^+^ cells and the lack of distance-dependency in CheA_S_^−^ cells, the response time was estimated in this simulation as the time required to decrease the CheY-P concentration below 3.2 µM after the inactivation of CheA activity (Figure 5B,C, arrows and dotted lines); the CW bias of the flagellar motor is known to change drastically around this concentration of CheY-P [22]. In the CheA_S_^+^ simulation, response times matched well with the distance-dependency of response times that we had measured experimentally (Figure 4C, gray line, and Figure S4E, blue line). The CheA_S_^−^ simulation explained the lack of distance-dependency in the experimentally measured response times (Figure 4C black line, and Figure S4E red line). These results indicate that the distance-dependency of the response time in CheA_S_^+^ cells and its lack in CheA_S_^−^ cells are correlated with the diffusion of CheY and CheY-P molecules and the area of phosphorylation of CheY and of dephosphorylation of CheY-P, respectively. Thus, in wild-type (CheA_S_^+^) cells, the polar localization of CheZ delays the decrease in CheY-P concentration depending on the distance from the receptor array. In contrast, in CheA_S_^−^ cells, the CheY-P concentration decreases at the same rate throughout the cell because CheZ is distributed uniformly throughout the cytoplasm.

By comparing the experimentally measured cellular response time to photoreleased serine for a motor near the receptor array (~0.1 µm2 from cell pole), the response time of CheA_S_^−^ cells was about 100 ms longer than that of CheA_S_^+^ cells (Figure 4C). To explain this difference in response time using the simulation, the rate constant of the phosphatase activity of CheZ in the receptor array (see Materials and Methods) must be set ~2.5-fold higher than that of CheZ in the cytoplasm (Figure 4C, black and gray lines). The higher activity of polar-localized CheZ is consistent with a previous report of the phosphatase activity of CheZ in the presence and absence of CheA_S_ in vitro [27]. Therefore, the assembly of CheZ within the receptor array also enhances its phosphatase activity in living *E. coli* cells.

## 4. Discussion

### 4.1. Mechanism for Coordination of Switching among Flagellar Motors on an E. coli Cell

To clarify the mechanism of intracellular signaling during chemotaxis in *E. coli*, we previously measured the coordination of rotational switching of two different flagellar motors on the same cell [13]. That study showed that two flagellar motors on the same cell coordinately switch their rotational direction in the absence of external stimuli. The switching time lag (∆τ_correlation_) of two coordinated motors depended on their relative distance from the receptor array at the cell pole. A mutant cell lacking the CheZ protein did not exhibit coordinated switching. Coordinated switching was also inhibited by the expression of a constitutively active mutant form of CheY, which mimics the CW rotation-stimulating function of wild-type CheY-P, regardless of its phosphorylation state [13,28]. These results suggested that fluctuations in CheY-P concentration regulate the coordination of switching between flagellar motors.

**Figure 6:**
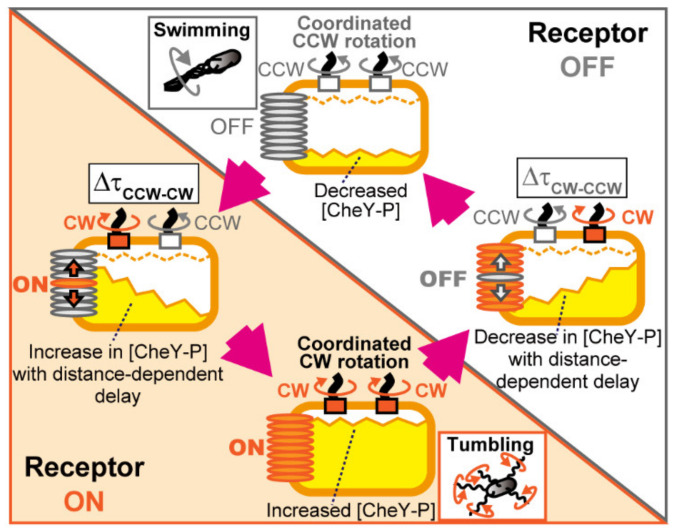
A model for intracellular signaling in an *E. coli* cell under steady-state conditions with no external stimuli. The orange-hatched and white-hatched triangles indicate times when the receptor array is active or inactive, respectively. When the receptor units (the receptor/CheW/CheA complexes) are in an inactive state (depicted as gray ellipses located at cell pole), flagellar motors on the same cell rotate CCW. When one or small numbers of the receptor units are activated (orange ellipse depicted in left cell), its activation is propagated through the receptor array and/or sub-arrays (multiple interconnected segments in the array) because of the cooperativity among the receptor units. The CheY-P concentration increases at the cell pole through the activity of CheA, and the increase propagates through the cytoplasm by diffusion with a delay dependent on the distance from the receptor array (yellow area within a cell). Therefore, two flagellar motors coordinately switch rotational direction from CCW to CW with a delay (left and bottom cells). On the other hand, when one or several of the receptor units is inactivated (gray ellipse depicted in right cell), its inactivation is propagated through the receptor array and/or sub-arrays because of the cooperativity among the receptor units. The CheY-P concentration is decreased at the cell pole through the activity of CheZ with a delay dependent on the distance from receptor array due to the diffusion of CheY-P molecules. Therefore, two flagellar motors coordinately reverse their rotational direction from CW to CCW with a delay (right and top cell). In the steady-state, the spontaneous blinking in activity of receptor array causes the fluctuation in CheY-P concentration that coordinates the switching of rotational direction among the flagellar motors.

In the present study, we asked how motor coordination changes when CheZ is uniformly distributed throughout the cell rather than localized at the receptor array. We found that time lags in CCW-CW switching (∆τ_CCW-CW_) increased as a function of the distance of the two motors from the pole containing the receptor array, both in the presence and absence of polar localization of CheZ (Figure 3). On the other hand, the distance-dependency of time lags in CW-CCW switching (∆τ_CW-CCW_) disappeared with the absence of polar localization of CheZ (in CheA_S_^−^ cells), whereas it was retained in the presence of polar localization of CheZ (in CheA_S_^+^ cells). Therefore, we propose that, in CheA_S_^+^ cells, dephosphorylation of CheY-P by polarly localized CheZ allows the CheY-P concentration to drop more rapidly at motors close to the receptor array. In CheA_S_^−^ cells with uniformly distributed CheZ, the CheY-P concentration decreases at the same rate at any distance from the receptor array. These results are consistent with the distance-dependency and distance-independency of response times to photoreleased serine in CheA_S_^+^ and CheA_S_^−^ cells, respectively (Figure 4C). CheA localizes at the cell pole in both CheA_S_^+^ and CheA_S_^−^ cells, so phosphorylation of CheY always occurs at a cell pole. Therefore, the CheY-P concentration increases more rapidly at motors close to the receptor array in both CheA_S_^+^ and CheA_S_^−^ cells, and in both cell types there is a delay in CCW-CW switching for motors distal from the receptor array relative to motors proximal to the array (Figure 6).

Recent theoretical studies have proposed that intrinsic motor-to-motor coupling caused by hydrodynamic interactions between motors is responsible for coordinated motor switching [29]. This possibility was explored in our previous investigation, but we did not see coordination of motors on different cells that were very close to one another [13]. We only saw coordination between motors on the same cell, indicating that hydrodynamic interaction between motors is not the main contributor to coordinated switching.

We simulated switching coordination between motors, assuming a steep sigmoidal relation of CW bias and switching frequency to CheY-P concentration, as shown by Cluzel et al. [22]. Thus, CW bias and switching frequency reflect CheY-P concentration (see Supplementary Methods). Switching coordination was seen in simulations when fluctuations in CheY-P concentration were taken into account (Figure S5A–C). In contrast, motors stochastically switched their rotational direction at a constant CheY-P concentration (Figure S5D–F). Another theoretical model from the Namba-Shibata group reproduced the strong coordination of rotational switching between two motors caused by fluctuations of the CheY-P concentration arising from spontaneous fluctuations in the kinase activity of the receptor array [30]. We conclude that fluctuation in the CheY-P concentration is the main contributor to the coordination of switching between motors on a cell.

### 4.2. Coordinated Motor Switching via Diffusive Signal Propagation from Receptor Arrays

As shown in the present study, both ∆τ_CCW-CW_ and ∆τ_CW-CCW_ increase as a function of the distance of the two motors from the cellular pole containing the receptor array in CheA_S_^+^ cells. The apparent diffusion coefficients estimated from these data were 9.3 and 9.6 µm^2^/s, respectively, in agreement with estimates for CheY-P obtained in previous studies [1,13,19]. The distance-dependency of ∆τ_CCW-CW_ in CheA_S_^+^ cells was explained well by a simulation that took into consideration the phosphorylation of CheY by polarly localized CheA, the dephosphorylation of CheY-P by polarly localized CheZ, and the diffusion of CheY and CheY-P molecules (Figure 5 and Figure S4). The distance-dependency of ∆τ_CCW-CW_ in CheA_S_^−^ cells with uniformly distributed CheZ was also explained by considering the phosphorylation of CheY by polarly localized CheA, the dephosphorylation of CheY-P by bulk-cytoplasmic CheZ, and the diffusion of CheY and CheY-P molecules. However, the distance-dependency of ∆τ_CW-CCW_ in CheA_S_^−^ cells was lost in this simulation because the uniformly distributed CheZ causes CheY-P levels to fall at the same rate throughout the cell. Therefore, fluctuation depends on the diffusion of CheY and CheY-P molecules and signal-producing and signal-destroying reactions in the receptor array (Figure 6).

To produce fluctuations in CheY-P concentration over timescales of several hundred milliseconds, a receptor array, which is composed of more than 10,000 protein molecules, would work as one single or several large signaling units, which is possible because of the high cooperativity derived from the network architecture [31]. Multiple interconnected segments in the receptor array (sub-arrays), which work independently of each other, and/or the entire receptor array, would spontaneously blink between active and inactive states in the absence of chemoeffectors. A theoretical model to explain the spontaneous blinking of array activity was proposed by the Namba-Shibata group [30]. Their model incorporated two states (active and inactive) of each receptor unit and cooperativity among the units constituting the array. Both CheB and CheY are phosphorylated by CheA when the receptor array is activated. In this situation, CheB-P demethylates the receptor to bring the receptor array into an inactive state. On the other hand, when the receptor array is inactive, methylation of the receptors promoted by the constitutive activity of CheR brings the array to an active state. Therefore, changes in the methylation level produced by the activity of CheB-P and CheR could affect the spontaneous blinking in the activity of the receptor array, as mentioned by Shimizu et al. [32]. The fluctuation in CheY-P concentration measured in a single cell through FRET between CheY-YFP and CheZ-CFP has also been reported for cells possessing or lacking CheR and CheB [33,34]. The sampling rate in the FRET experiments (1–0.2 Hz) is very different from our high-speed imaging (~1250 Hz), so further experiments are required to verify that fluctuations in CheR and CheB activity lead to fluctuations in CheY-P production.

### 4.3. Behavioral and Evolutionary Implications of the Blinking Array Model

Despite the stochastic nature of the run-and-tumble swimming pattern of *E. coli* cells, it was recently reported that a swimming cell expressing wild-type CheY coordinates rotational switching between motors. This coordination of switching in swimming cells was not observed in the presence of a constitutively active mutant form of CheY [35]. These results indicate that the coordination of motor switching occurs in a swimming cell, and the run-and-tumble behavior is caused not only through stochastic switching of the flagellar motors but also coordination of motor switching regulated by fluctuations in CheY-P concentration (Figure 6). Turner et al. reported that the distribution of directional changes from run to run during a tumble is narrow and biased in the forward direction when a smaller number of flagella are unwound from the flagellar bundle, whereas the distribution is wide when a larger number of flagella are unwound from the bundle [36]. The coordination of switching among flagellar motors leads to the unwinding of a larger number of flagella from the bundle during a tumble; therefore, the cell undergoes a bigger change in the direction of swimming. Fluctuations in CheY-P concentration under steady-state conditions could be a strategy that enables *E. coli* to explore its environment more widely by regulating run-and-tumble swimming through the coordinated switching of motors.

*Bacillus subtilis*, a rod-shaped Gram-positive bacterium evolutionarily very distant from *E. coli*, is also peritrichously flagellated, has a polar receptor array, and carries out chemotaxis by biasing the run-and-tumble swimming pattern [37]. However, the chemotaxis system of *B. subtilis* is quite different from that of *E. coli*; CheY-P induces CCW flagellar rotation, and thus promotes runs, and chemoattractants cause receptors to stimulate the activity of CheA in phosphorylating CheY. CheY-P is dephosphorylated at the cytoplasmic face of the flagellar basal body by the FlaY protein instead of by CheZ. Therefore, the flagellar motor acts as a sink to deactivate the run signal. Are the flagellar motors of *B. subtilis* coordinated? If so, how do they achieve coordinated switching? It will be interesting to model the dynamics of CheY-P in *B. subtilis* to see whether they also lead to the coordination of multiple flagella. *B. subtilis* might be using a very different paradigm from the one that is used by *E. coli*.

A swimming bacterium hydrolyzes ATP to produce CheY-P under steady-state conditions. The cell must remain in a state of run-and-tumble readiness to respond quickly when chemoeffectors are encountered. There must be a substantial advantage to maintain the chemotaxis system on high alert rather than to decrease energy consumption by keeping it in a resting mode. Here, we report how this system is organized in *E. coli*. The challenge remains to determine whether and how other bacteria may have evolved different CheY-dependent mechanisms to coordinate locomotor behavior.

## Supplementary Materials

The following are available online at https://www.mdpi.com/2218-273X/10/11/1544/s1, Supplementary Methods, Table S1: Bacterial strains and plasmids, Figure S1: The switching coordination between two motors in CheA_S_^−^ cell measured at 1255 fps, Figure S2: The switching coordination between two motors in a mutant cell, which has the substitution for F98S in CheZ (CheZ(F98S) cell), Figure S3: Schematic diagram of the measurement system to measure the cellular response time to serine signal photoreleased from caged serine, Figure S4: Simulation for the change in CheY-P concentration in the presence and absence of the polar localization of CheZ, Figure S5: Simulation for the coordination of the directional switching between motors [38,39].
