# Supplementary material for: Fluctuations in Intracellular CheY-P Concentration Coordinate Reversals of Flagellar Motors in E. coli
Source: Biomolecules. 2020 Nov 12;10(11):1544. doi: 10.3390/biom10111544 (PMC7696710; doi:10.3390/biom10111544)
Supplement: Supplementary file 1 [file biomolecules-10-01544-s001.pdf]

biomolecules

MDPI

Fluctuations in Intracellular CheY-P Concentration Coordinate Reversals of Flagellar Motors in *E. coli*.

Yong-Suk Che, Takashi Sagawa, Yuichi Inoue, Hiroto Takahashi,Tatsuki Hamamoto, Akihiko Ishijima and Hajime Fukuoka

Supplementary Methods

Cell growth

LB broth (1% bactotryptone, 0.5% yeast extract, 0.5% NaCl) was used for culture growth, transformations, and plasmid isolation. Tryptone broth (TB) (1% bactotryptone, 0.5% NaCl) was used to grow cells for measurements of motor rotation. For the monitoring of the rotation of flagellar motors on cells that are wild type for chemotaxis (CheAS+), EFS031 cells [13] harboring pTH2300, which encodes *motA* and *motB*, and pHFGW2, which encodes *gfp-cheW*, were used. For the monitoring of the rotation of flagellar motors on CheAS- cells, EFS043 cells harboring pTH2300 and pHFGW2 were used. For the monitoring of the rotation of flagellar motors on cells that overproduce CheAS, EFS031 cells harboring pTH2300 and pFYC2, which encode *gfp-cheW* and *cheA*(M98L), were used. For the monitoring of the rotation of flagellar motors on *cheZ*(F98S) mutant cells, EFS049 cells harboring pTH2300 and pHFGW2 were used. These cells were grown in TB containing 30 μM IPTG, 0.002% arabinose, 25 μg/mL chloramphenicol, and 50 μg/mL ampicillin at 30 °C for 5.25 h. To observe the polar localization of CheZ-GFP in the presence of wild-type *cheA* gene, EFS032 cells [13] harboring pTH2400, which encodes *motA* and motB, and pHF6503, which encodes *cheZ-egfp*, were used. To observe the localization of CheZ-GFP in CheAS- cells, EFS045 cells harboring pTH2400 and pHF6503 were used. To observe the localization of CheZ(F98S)-GFP in CheAS+ cells, EFS032 cells harboring pHF6503(F98S), which encodes *cheZ*(F98S)-*egfp* and pTH2400 were used. These cells were grown in TB containing 10 μM IPTG, 0.01% arabinose, 25 μg/mL chloramphenicol, and 50 μg/mL ampicillin at 30 °C for 5.25 h.

Measurement of rotation of multiple flagellar motors

Cells were prepared by a method similar to that described in our previous report [13]. One ml of cell culture was centrifuged, and the pellet was suspended in 1 ml of 10NaMB (10 mM potassium phosphate buffer, pH 7.0; 0.1 mM EDTA-2K, pH 7.0; 10 mM NaCl, 75 mM KCl). The cell suspension was centrifuged, and the pellet was suspended in ~300 μl of 10NaMB. The cell suspension was loaded into the sample chamber made by 18 × 18 and 24 × 50 mm coverslips with a spacer and incubated for 15 min to allow the cells to attach to the coverslip. The inside of the sample chamber was gently perfused with additional 10NaMB to remove the remaining unattached cells. A suspension of polystyrene beads, with a diameter of 0.5 μm, was injected, and the mixture was incubated for 15 min to allow the beads to attach to the flagellar filaments. The space between coverslips was gently perfused again with additional 10NaMB to remove unattached beads.

The rotation of the beads was measured using the same microscopic system that we reported previously [19]. The beads were observed under phase-contrast microscopy (IX71; Olympus, Tokyo, Japan). The phase-contrast images of the beads through the objective lens (UPlanFl 40× NA 0.75 Ph2; Olympus, Tokyo, Japan) were recorded with a high-speed charge-coupled device (CCD) camera (IPX-VGA210LMCN; Imperx, Boca Raton, FL) at 1250 or 1255 frames/s. Each captured image was transferred via a frame-grabber card (NI PCIe-1429; National Instruments, Austin, TX) to a computer for image analysis. This high-speed CCD camera was controlled by the measurement software Real Time Video Nanometry (RTVN), which we developed using LabVIEW 2009 (National Instruments, Austin, TX). The phase-contrast images of the beads were fitted by a two-dimensional Gaussian function for every sampling frame, and the position of a bead was expressed as X and Y coordinates of a fitted Gaussian curve. The bead position was approximated by an ellipse function every 500

frames, and the bead position was corrected to approximate a perfect circle centered on the origin. The rotation angle was calculated for every two sampling frames, and a time trace of the angular velocity, rotational velocity, and rotational direction were estimated by repeating this process every video frame.

To observe the location of GFP-CheW, a blue laser beam (wavelength (λ) 488 nm) (Sapphire 488-20-SV; Coherent, Hercules, CA) was reflected by a dichroic mirror (FF495 Di02; Semrock, USA) and focused on the back focal plane of the objective lens. The fluorescence, which was passed through the dichroic mirror and the emission filter (FF01-520/35, Semrock, USA), was focused on a second CCD camera (DMK23G618; The Imaging Source, Bremen, Germany) (Figure 1A). After recording the bead rotation with the high-speed CCD camera, the fluorescence image of GFP-CheW and the phase-contrast images of bead and cell were recorded at 120 frames/s with the second CCD camera to monitor the position of the bead on the cell. The distance between the fluorescent focus at the cell pole derived from the GFP-CheW fluorescence and the rotational center of the rotating bead was measured and defined as the distance from the receptor array to motor.

Measurement of the response time to the instantaneously applied photoreleased serine

Cell preparation and the attachment of polystyrene beads (ϕ = 0.5 μm) to flagellar stubs were performed as described above. To remove unattached beads and apply caged serine, the space inside the flow chamber was then gently perfused twice with additional 10NaMB containing the appropriate concentration of caged serine. The caged serine that we synthesized in our previous study was used [19].

The microscopic system and the conditions for photoreleasing serine from caged serine were the same as in our previous study [19] (Figure S3). Caged serine surrounding an *E. coli* cell in a microscopic field was photolyzed by irradiation with a violet laser beam (wavelength (λ) 405 nm) (KBL-90C-A; Kimmon Koha, Tokyo, Japan, or OBIS 405-50 LX; Coherent, Hercules, CA) for 80 ms. For the photorelease of serine, the violet laser beam was reflected by a dichroic mirror (FF495 Di02; Semrock, USA) and focused on the back focal plane of the objective lens (UPlanFl 40× NA 0.75 Ph2; Olympus, Tokyo, Japan). The diameter of the irradiated area was adjusted to 32 μm. The violet laser beam was uniformly applied to the irradiated area, and the energy density of the laser beam in the irradiated area was 370 $mW \cdot mm^{-2}$. The rate constant for the photolysis of caged serine in the irradiated area was 0.16 s^{-1}. The serine stimulus was applied to each cell only once. The rotational velocity and direction were estimated in real time through the RTVN from the position of the bead-mass center. When RTVN detected the switching of the flagellar motor from the CCW to CW, it sent a 5 V TTL signal from an D/A converter (NI USB-6212; National Instruments, Austin, TX) to an electric shutter driver (VCM-D1; Uniblitz, San Diego, CA) to open a mechanical shutter positioned in front of the laser beam for 80 ms (UHS1 ZM 2; Uniblitz, San Diego, CA). The distance between the polar receptor array and the flagellar motor was quantified using the same method described above.

Simulation for coordination of the directional switching between motors

The time traces of rotational direction of the two motors were simulated, and correlation analysis was performed between the simulated time traces of the rotational direction in the presence and absence of the fluctuation in CheY-P concentration.

We hypothesized that the motor switching occurs through the following reaction scheme:

$$CCW \underset{k_{CCW}}{\overset{k_{CW}}{\rightleftarrows}} CW$$

where CCW and CW are the states of CCW and CW rotation, respectively, and k_{CW} and k_{CCW} are the rate constants for CCW-to-CW and CW-to-CCW switching, respectively. We also assumed that CCW and CW durations show exponential distribution:

$$f_{CCW} = k_{CW} \cdot e^{-k_{CW} \cdot t} \quad (1)$$

$$f_{CW} = k_{CCW} \cdot e^{-k_{CCW} \cdot t} \quad (2)$$

where f_{CCW} is distribution of CCW duration, f_{CW} is the distribution of the CW duration, t is a time. In this reaction, the averages of CCW and CW duration are $\frac{1}{k_{CW}}$ and $\frac{1}{k_{CCW}}$, therefore the relation of $\frac{1}{k_{CW}}$, $\frac{1}{k_{CCW}}$, and CW bias (α) is expressed as follows;

$$\frac{1}{k_{CW}} : \frac{1}{k_{CCW}} = (1-\alpha) : \alpha$$

$$\frac{1}{k_{CW}} = \frac{1-\alpha}{\alpha} \cdot \frac{1}{k_{CCW}} \quad (3)$$

In addition, relation of $\frac{1}{k_{CW}}$, $\frac{1}{k_{CCW}}$, and switching frequency (F) is expressed as follows;

$$\frac{1}{k_{CW}} + \frac{1}{k_{CCW}} = \frac{2}{F} \quad (4)$$

From Equation 3 and Equation 4, k_{CW} and k_{CCW} are expressed by the function of CW bias (α) and switching frequency (F) as follows;

$$k_{CW} = \frac{F}{2 \cdot (1-\alpha)} \quad (5)$$

$$k_{CCW} = \frac{F}{2\alpha} \quad (6)$$

$$0 < \alpha < 1$$

Cluzel *et al.* [22] reported a steep sigmoidal relation of CW bias (α) and CheY-P concentration ([YP]), and a relation was expressed by Hill function;

$$\alpha = \frac{[\mathrm{YP}]^{\mathrm{n}}}{\mathrm{K_D}^{\mathrm{n}} + [\mathrm{YP}]^{\mathrm{n}}} \quad (7)$$

where n is the Hill coefficient and K_D is the dissociation constant. This paper [22] also reported a relation of the switching frequency (F) qualitatively behaves $F = A \cdot \frac{\partial(CW\ bias)}{\partial[YP]}$, and F is also fitted by the first derivative of the Hill function with respect to CheY-P concentration. Therefore,

$$\mathrm{F} = \mathrm{A} \cdot \frac{\partial(\mathrm{CW\ bias})}{\partial[\mathrm{YP}]} = \mathrm{A} \cdot \frac{\mathrm{n} \cdot \mathrm{K_D}^{\mathrm{n}} \cdot [\mathrm{YP}]^{\mathrm{n}-1}}{(\mathrm{K_D}^{\mathrm{n}} + [\mathrm{YP}]^{\mathrm{n}})^2} \quad (8)$$

where A is a constant. From Equation 5, 7, and 8,

$$k_{CW} = \frac{F}{2 \cdot (1-\alpha)} = \frac{\mathrm{A} \cdot \mathrm{n} \cdot [\mathrm{YP}]^{\mathrm{n}-1}}{2 \cdot (\mathrm{K_D}^{\mathrm{n}} + [\mathrm{YP}]^{\mathrm{n}})} \quad (9)$$

From Equation 6, 7, and 8,

$$k_{CCW} = \frac{F}{2\alpha} = \frac{\mathrm{A} \cdot \mathrm{n} \cdot \mathrm{K_D}^{\mathrm{n}}}{2 \cdot [\mathrm{YP}] \cdot (\mathrm{K_D}^{\mathrm{n}} + [\mathrm{YP}]^{\mathrm{n}})} \quad (10)$$

The KD and Hill coefficients were estimated as 3.1 M and 10.3 by Cluzel et al. [22]; therefore, these values were also used in this paper. We set the value of the constant A to 2, which is a value that reproduces the fitting curve for the switching frequency shown by Cluzel et al. [22].

Therefore, distributions of the CCW duration (f_{CCW}) at a certain CheY-P concentration could be estimated by using Equation 1 and Equation 9, and distributions of CW duration (f_{CW}) at a certain CheY-P concentration could be estimated by using Equation 2 and Equation 10.

When the fluctuation of CheY-P concentration was applied, for simplification, we hypothesized that the CheY-P concentration ([YP]) fluctuates between two states, a low-concentration state ($[YP]_{Low}$) and a high-concentration state ($[YP]_{High}$) as follows;

$$[YP]_{Low} \underset{k_-}{\overset{k_+}{\rightleftarrows}} [YP]_{High} \tag{11}$$

where k_+ and k_- are the rate constants for $[YP]_{Low}$-to-$[YP]_{High}$ transition and $[YP]_{High}$-to-$[YP]_{Low}$ transition. Distributions of the durations in $[YP]_{Low}$ and $[YP]_{High}$ were assumed to be a single exponential, and k_+ and k_- were set to 1 s^{-1} and 2 s^{-1}, respectively. The CheY-P concentration went back and forth between 2.1 µM ($[YP]_{Low}$) and 4.1 µM ($[YP]_{High}$) centering on 3.1 µM (K_D).

Table S1. Bacterial strains and plasmids.

	Description	Reference
Strains		
RP437	Wild-type for motility and chemtaxis	[14]
EFS031	RP437 Δ*motA* Δ*motB fliC-sticky*	[13]
EFS032	RP437 Δ*motA* Δ*motB* Δ*cheZ fliC-sticky*	[13]
EFS041	RP437 Δ*motA* Δ*motB cheA*(M98L)	This work
EFS043	RP437 Δ*motA* Δ*motB cheA*(M98L) *fliC-sticky*	This work
EFS045	RP437 Δ*motA* Δ*motB cheA*(M98L) Δ*cheZ fliC-sticky*	This work
EFS049	RP437 Δ*motA* Δ*motB cheZ*(F98S) *fliC-sticky*	This work
Plasmids		
pMMB206	Cm^r $P_{tac\text{-}lac}$	[38]
pBAD24	Ap^r P_{BAD}	[39]
pTH2300	*motAmotB* in pMMB206	[13]
pTH2400	*motAmotB* in pBAD24	This work
pHFGW2	*egfp-cheW* in pBAD24	This work
pFYC2	*egfp-cheW cheA*(M98L) in pBAD24	This work
pHF6503	*cheZ-egfp in pMMB206*	This work
pHF6503(F98S)	*cheZ*(F98S)-*egfp in pMMB206*	This work

Ap^r, ampicillin-resistant; Cm^r, chloramphenicol-resistant; P_{lac}, *lac* promoter; P_{tac}, *tac* promotor; P_{BAD}, *araBAD* promoter.

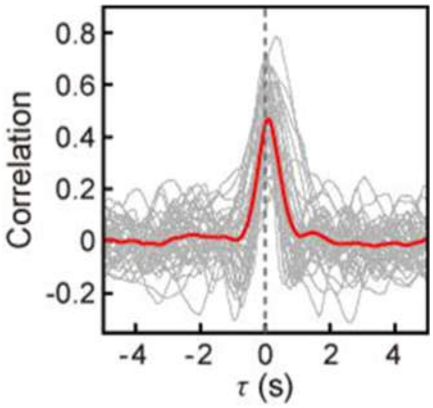

Figure S1. The switching coordination between two motors in $CheAs^-$ cell measured at 1,255 fps. Gray lines indicate an individual correlation analysis for cells. The analysis was performed on 41 cells with monopolar or bipolar localization of GFP-CheW, and on the cells where the expression of GFP-CheW was not observed. The traces of 39 cells detected for switching coordination are shown. The red line indicates the average traces of the correlation analyses from 39 cells.

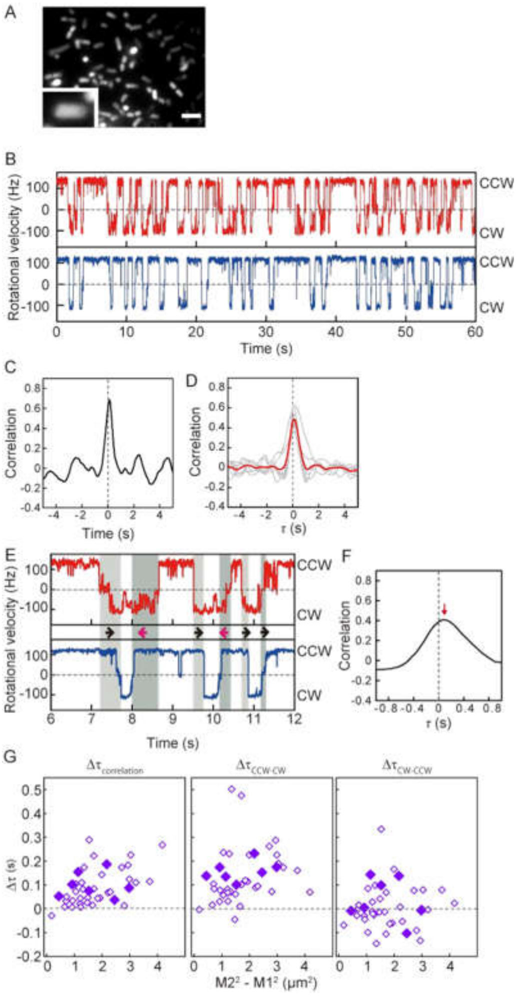

Figure S2. The switching coordination between two motors in a mutant cell, which has the substitution for F98S in CheZ (CheZ(F98S) cell). (**A**) Observation of CheZ(F98S)-EGFP in the *cheZ* deleted cell. CheZ(F98S)-EGFP was diffused in the cytoplasm, and polar localization was not observed. Bar, 3.0 m. (**B**) The time traces of the rotational directions of the proximal motor 1 (red) and the distal motor 2 (blue) on a CheZ(F98S) cell. Two motors on this cell coordinately switch their rotational direction. (**C**) Cross correlation profile between motors 1 and 2, which are depicted in Figure S2B. Correlation analysis showed a near 0-s peak. (**D**) The gray lines indicate an individual correlation analysis for CheZ(F98S) cells (seven cells). The red line indicates the average trace of the correlation analyses from seven cells. These results indicate that multiple flagellar motors in a CheZ(F98S) cell coordinately switch their rotational direction, similar to CheA$_S$ cell (Figure 2); therefore, the polar localization of CheZ itself is not essential for the coordinated switching among flagellar motors. (**E**) The rotational direction of motors 1 (red) and 2 (blue) of a CheZ(F98S) cell, shown in Figure S2B, over a short time period. For the CCW-to-CW switching, motor 1, which was closer to the chemoreceptor array, preceded motor 2, which was farther away from the array. On the other hand, CW-to-CCW switching of the motor closer to the receptor array did not always precede the more-distant motor. Forward arrows indicate that the switching of motor 1 preceded that of motor 2, and reverse arrows indicate that the switching of motor 1 was delayed relative to the switching of motor 2. (**F**) Magnified traces of the correlation profile that are shown in Figure S2C. The red arrow indicates the time of the peak correlation (+88 ms). (**G**) The relationship between the value and [$M2^2$-$M1^2$] for the CheZ(F98S) cells. Left shows the relation for $\Delta_{correlation}$ estimated from the peak of the correlation profile. Middle and right show the relation for $\Delta_{CCW\text{-}CW}$ and $\Delta_{CW\text{-}CCW}$, respectively. This cell line also expressed GFP-CheW, and the correlation analyses were made based on the motor closer to the polar chemoreceptor array monitored by the localization of GFP-CheW. Closed purple squares indicate the relation for a CheZ(F98S) cells. Opened purple squares indicate the CheA$_S^-$ cells shown in Figure 3D.

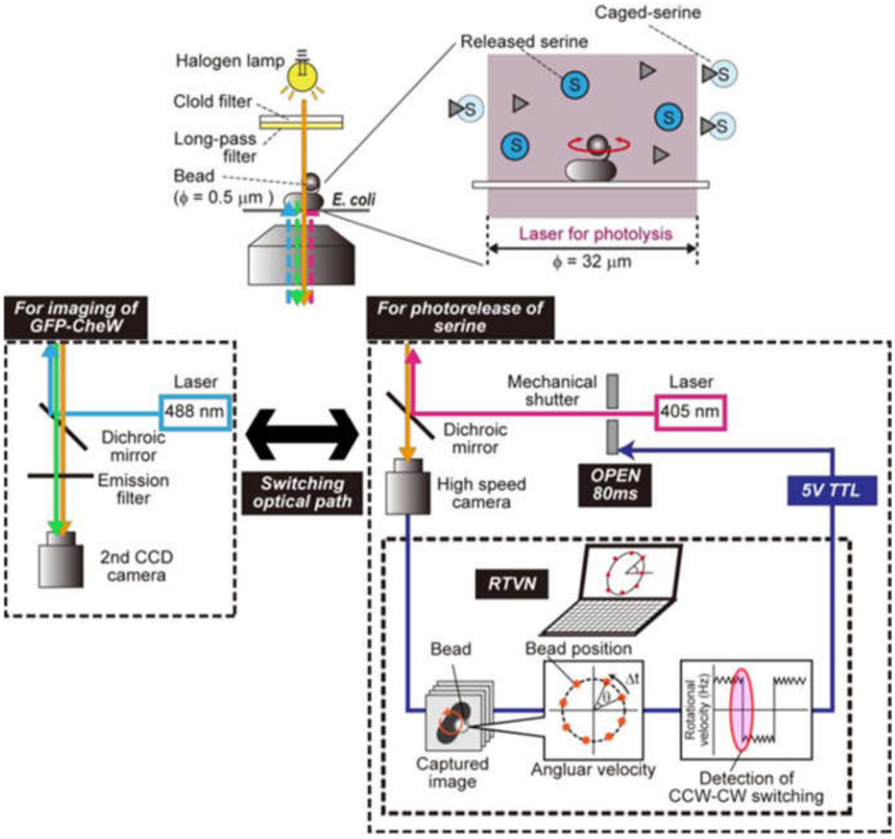

Figure S3. Schematic diagram of the measurement system to measure the cellular response time to serine signal photoreleased from caged serine. The cell was stuck to a coverslip, and polystyrene beads (= 0.5 m) were attached to the sticky flagellar stubs. The phase-contrast image of each bead was recorded with a high-speed CCD camera (1255 frames/s). A violet laser beam was focused on the back focal plane of the objective lens for the photolysis caged serine. The diameter of the irradiated area of the laser was adjusted to 32 m. RTVN captures the phase contrast image of the beads through a high-speed CCD camera and estimates the rotational direction in real time by calculating the angular velocity from the position of each bead. When RTVN detected the rotational switching of the flagellar motor from the CCW to CW direction, RTVN outputs 5V TTL to mechanical shutter, which was positioned on the optical axis of violet laser. As a result, the mechanical shutter opens for 80 ms and the violet laser was irradiated. To observe the localization of GFP-CheW, a blue laser beam was focused on the back focal plane of the objective lens. The fluorescence was passed through the dichroic mirror and the emission filter was focused on a second CCD camera. After recording the bead rotation with the high-speed CCD camera, the fluorescence image of GFP-CheW and the phase-contrast images of the bead and cell were recorded at 120 frames/s with the second CCD camera to monitor the position of the receptor array and the bead on the cell.

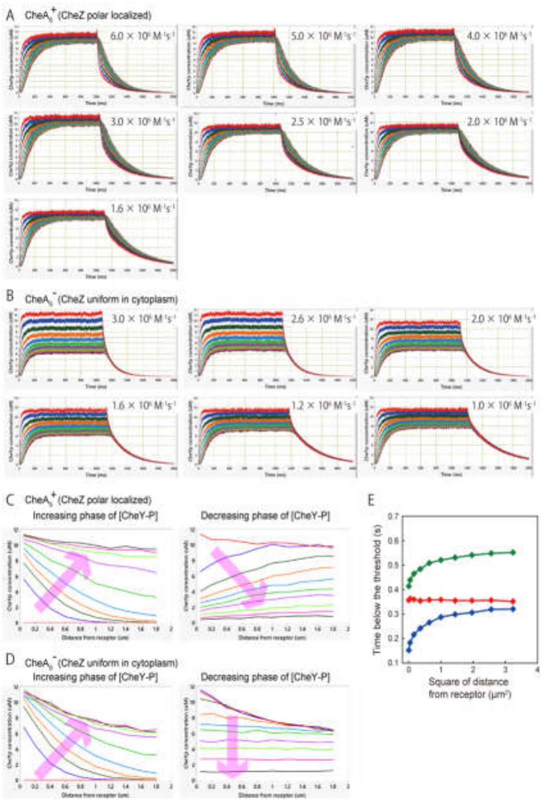

Figure S4. Simulation for the change in CheY-P concentration in the presence and absence of the polar localization of CheZ. The method for the simulation is described in the Materials and Methods in the main text. (**A**) Simulation for the CheA$_S^+$ cell when different k3 of polar localized CheZ was applied. The k3 values are depicted in the upper right corner of each graph. The distances between the cell pole and motor are 0.05 μm (red), 0.2 (blue), 0.4 (dark green), 0.6 (orange), 0.8 (cyan), 1.0 (green), 1.2 (violet), 1.4 (yellow green), 1.6 (magenta), and 1.8 (black), respectively. The simulation was carried out 13 times (k3 = 4.0 × 106 M-1 s-1) or 10 times (other k3 values), and the average traces for each k3 values are shown in the graph. (**B**) Simulation for CheA$_S$ cell when different k3 values of cytoplasmic CheZ are applied. Distances from the cell pole and color for graphs are the same as that of the CheA$_S^+$ cell. The simulation was carried out 10 times in each k3 condition and average traces were shown in the graph. (**C**) Relation of the distance from the polar receptor array and the CheY-P concentration for the CheA$_S^+$ cell estimated from the simulation shown in Figure S4A (k3 = 4.0 × 106 M-1 s-1). Left: increasing phase of CheY-P concentration after the activation of CheA. The relation between the simulated time and colors is as follows: 0 ms (red), 10 ms (blue), 20 ms (dark green), 30 ms (orange), 50 ms (turquoise), 100 ms (green), 200 ms (purple), 300 ms (yellow-green), 400 ms (magenta), and 500 ms (black). Right: decreasing phase of CheY-P concentration after the inactivation of CheA. The relation between the simulated time and colors as follows; 1000 ms (red), 1050 ms (blue), 1100 ms (dark green), 1150 ms (orange), 1200 ms (turquoise), 1250 ms (green), 1300 ms (purple), 1400 ms (yellow-green), 1500 ms (magenta), and 1600 ms (black). (D) Relation for the distance from polar receptor array and CheY-P concentration for the CheA$_S$ cell estimated from the simulation shown in Figure S4B (k3 = 1.6 × 106 M-1 s-1). Left: increasing phase of the CheY-P concentration. The relation between the simulated time and colors are the same for CheA$_S^+$ cell. Right: decreasing phase of the CheY-P concentration. The relation between the simulated time and colors as follows; 1000 ms (red), 1120 ms (blue), 1140 ms (dark green), 1160 ms (orange), 1180 ms (turquoise), 1200 ms (green), 1250 ms (purple), 1300 ms (yellow-green), 1400 ms (magenta), and 1600 ms (black). (E) Simulated relationship between the response time and the square of the distance from the cell pole. Lines indicate the time spent decreasing the CheY-P concentration below 3.2 μM after the inactivation of CheA's activity estimated from the simulation of the change in the CheY-P concentration (Figure S4A and S4B). Blue and green lines indicate the simulated relations for the CheA$_S^+$ cell (polar localized CheZ) when k3 are 4 × 106 and 1.6 × 106 M-1 s-1, respectively. The red line indicates the simulated relation for the CheA$_S$ cell (cytoplasmic CheZ) when k3 is 1.6 × 106 M-1 s-1.

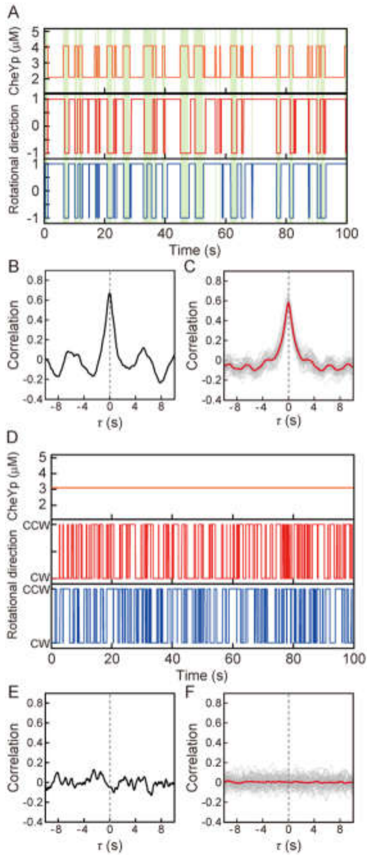

Figure S5. Simulation for the coordination of the directional switching between the motors. The time traces of the rotational direction of the two motors were simulated and a correlation analysis was performed between the simulated time traces of the rotational direction in the presence and absence of the fluctuation in the CheY-P concentration. Method The method for the simulation is described in the Supplemental Methods. (**A**) The typical switching behavior of the two motors when the fluctuation in the CheY-P concentration was applied. Top: the time trace of the fluctuation in the CheY-P concentration. The CheY-P concentration went back and forth between 2.1 and 4.1 μM, centering on 3.1 μM. Middle and bottom:, the simulated time traces of the rotational directions of two different motors when the change in CheY-P concentration was applied, as shown in Figure S5A, top. +1, CCW rotation. -1, CW rotation. Green hatches indicate the time periods during which the CheY-P concentration increased. As shown in Figure S5A, two motors coordinately switch the rotational direction in response to the change in CheY-P concentration. (**B**) Cross correlation profile for the simulated time traces of the rotational directions of the two motors, as shown in Figure S5A. Correlation analysis showed a peak near 0 seconds. (**C**) Gray lines indicate an individual correlation analysis for the simulated time traces of the rotational directions of the two different motors in the presence of the fluctuation in the CheY-P concentration (50 trials). Red The red line indicates the average trace of the correlation profiles from 50 trials. (**D**) The typical switching behavior of the two motors when the CheY-P concentration was constant at 3.1 μM. Top: the time traces of the CheY-P concentration. Middle and bottom: the simulated time traces of the rotational directions of the two different motors when the constant CheY-P concentration (Figure S5D, top) was applied. (**E**) Cross correlation profile for the simulated time traces of the rotational directions of the two motors shown in Figure S5D. Correlation analysis did not show a peak near 0 seconds. (**F**) Gray lines indicate an individual correlation analysis for the simulated time traces of the rotational directions of two different motors when the CheY-P concentration was constant at 3.1 μM (50 trials). Red The red line indicates the average trace of the correlation profiles from 50 trials. Average The average trace and all individual traces did not show a peak near 0 seconds. When the CheY-P concentration was constant, each motor stochastically and independently switches rotational direction at a given CheY concentration and did do not show the switching coordination. Therefore, the fluctuation in CheY-P concentration would be required to reproduce the switching coordination between two different motors on the same cell.
